# Minimal invasive annulotomy for induction of disc degeneration and implantation of poly (lactic-co-glycolic acid) (PLGA) plugs for annular repair in a rabbit model

**DOI:** 10.1186/s40001-016-0202-4

**Published:** 2016-02-29

**Authors:** Long Xin, Chun Zhang, Fuhua Zhong, Shunwu Fan, Wei Wang, Zhenbin Wang

**Affiliations:** Clinical Laboratory, Department of Orthopedics, Tongde Hospital of Zhejiang Province, Hangzhou, China; Department of Orthopaedic Surgery, Sir Run Run Shaw Hospital, School of Medicine, Zhejiang University, Hangzhou, China; Department of Polymer Science and Engineering, Zhejiang University, Hangzhou, China; Department of Polymer Materials Science and Engineering, Tianjin University, Tianjin, China; Clinical Laboratory, Department of Spine Surgery, The Fourth Affiliated Hospital of Xinjiang Medical University, Urumqi, 830000 China

**Keywords:** Minimal annulotomy, Mini-trephine, Disc degeneration, PLGA plug, T-cannula, Biological repair

## Abstract

**Background:**

The rabbit disc model is useful for the study of intervertebral disc (IVD) degeneration and experimental therapeutic interventions. The annulotomy-induced disc models present several drawbacks, particularly an excessive disruption of disc integrity and a rapidly disc degeneration; therefore, this study sought to establish a minimal invasive annulotomy for induction of disc degeneration model, combined to annulus repair using implantation of a PLGA (poly lactic-co-glycolic acid) plug.

**Methods:**

New Zealand white rabbits (*n* = 24) received annular injuries in three discs (L3/4, L4/5 and L5/6). The experimental discs were randomly assigned to four groups: (a) annular defect with a 1.8 mm diameter mini-trephine; (b) annular puncture by 16G needle; (c) annular defect with a PLGA plug implanted by press-fit fashion; (d) uninjured L2/3 disc served as control. Postsurgical x-ray, MRI examination, and real-time PCR analysis were performed at 1, 3 and 6 months. Gross morphology and histology were evaluated at postoperative 6 months.

**Results:**

Radiographic examinations showed a slow, progressive disc space narrowing and a significant degree of disc degeneration on MRI grade in the injured discs at 6 months in all rabbits. Histological examinations and aggrecan, Col1A1, Col2A1 and matrix metalloprotease (MMP)-3 mRNA expression confirmed the disc degeneration, supporting the imaging results. The PLGA implantation reduced the marked loss of T2-weighted signal intensity seen at MRI in the injured discs and slowly decreased the disc height index (DHI) over the follow-up period. HE/Safranin O staining showed that annular defect was replaced by regenerated connective tissue with significant loss of proteoglycan content.

**Conclusions:**

The minimally invasive approach for the creation of annular defects by an appropriately sized mini-Trephine is a suitable option for the study of disc degeneration in a rabbit model. Implantation of a suitable PLGA plug induced a successful repair of the annulus fibrosus within the degenerated disc, and retarded the degenerative process in the annular injury model.

## Background

The annular fissure and altered spinal mechanics are considered to play a critical role in initiation of degenerative changes within intervertebral disc (IVD) [1]. If annular fissures remain untreated, chronic discogenic low back pain may occur in 26–42 % of patients with internal disc disruption [2, 3]. Thus, there is an urgent need of experimental animal models that simulate the human disc degenerative processes and enable the evaluation of novel therapeutic procedures for the repair of annular lesions.

Numerous animal models of annulus fibrosus (AF) injury have been described for the inducing of IVD degeneration and the testing of disc regeneration therapies [4, 5]. Full-thickness AF injury using stab or puncture technique is popular and easily reproducible models in small animals [6–9]. These procedures, however, carry the risk of serious side effects such as undesirable osteophyte and overly rapid disc degeneration processes that less closely reflect the chronic course of clinical setting [10–12].

Larger animal models are focused on accomplishing the partial-thickness (annular rim lesion) or total annulotomy in imitation of disc degeneration [13–17]. The annulotomy-induced models also present some relevant drawbacks, for example a pronounced structural disruption of the disc, a rapidly decreasing disc height, acceleration of spinal biomechanics, increase of segmental instability, and relatively high mortality [18–21]. To date, rabbit disc models have been frequently used for the study of IVD degeneration and experimental therapeutic interventions due to their cost-effectiveness and acceptable similarities to the pathological process of human disc degeneration [4, 5]. Interestingly, there have been only limited attempts to induce models with a slow and moderate disc degeneration using a less invasive annulotomy in the rabbit model. A goal of this study was therefore to perform a minimally invasive annulotomy with limited annulus trauma. For this purpose, an appropriately sized mini-trephine was designed for the partial removal of the AF and consequent induction of IVD degeneration.

An intact and functional AF is a key to retard further degeneration of the IVD. Because of the limited intrinsic healing capability of the AF, effective annular repair may significantly improve the treatment outcome of degenerative spinal diseases [22, 23]. Tissue-engineered constructs have been recently applied to regenerate the AF and restore annulus structural integrity [23–25]. Poly lactic-co-glycolic acid (PLGA), a known biodegradable and biocompatible polymer, has recently received a great amount of interest. PLGA constructs have the mechanical property to reduce disc degeneration and allow a three-dimensional scaffolding for successful disc reconstruction [26–31]. However, the results of the biological repair are sometimes disappointing, with early loss of implanted materials through the annulus defects generated by the annulotomy itself [32–35]. Thus, a second goal of this study was to evaluate the results of annular repair by polymeric materials and minimize the risk of implant extrusion. For this purpose, a suitable T-cannula was used to insert the PLGA plug and close the defect by press-fitting. The hypothesis for this research was that minimally invasive annulotomy by an appropriately sized mini-trephine would induce the loss of disc height and cause degenerative changes, and that implantation of the PLGA plugs would improve the reparative capacity in the annulus-injured rabbit model.

## Methods

### Animals

A total of 24 New Zealand rabbits, age 8 months, weight 3.16 ± 0.2 kg, were supplied by the Laboratory Animal Center of Zhejiang province. Protocols were conducted in accordance with the Guidance for the Care and Use of Laboratory Animals, as formulated by the Ministry of Science and Technology of the People’s Republic of China. The rabbits were randomly allocated to 0.5-, 1-, 3- and 6-month survival groups (*n* = 6 in each group).

### Instruments

**Fig. 1:**
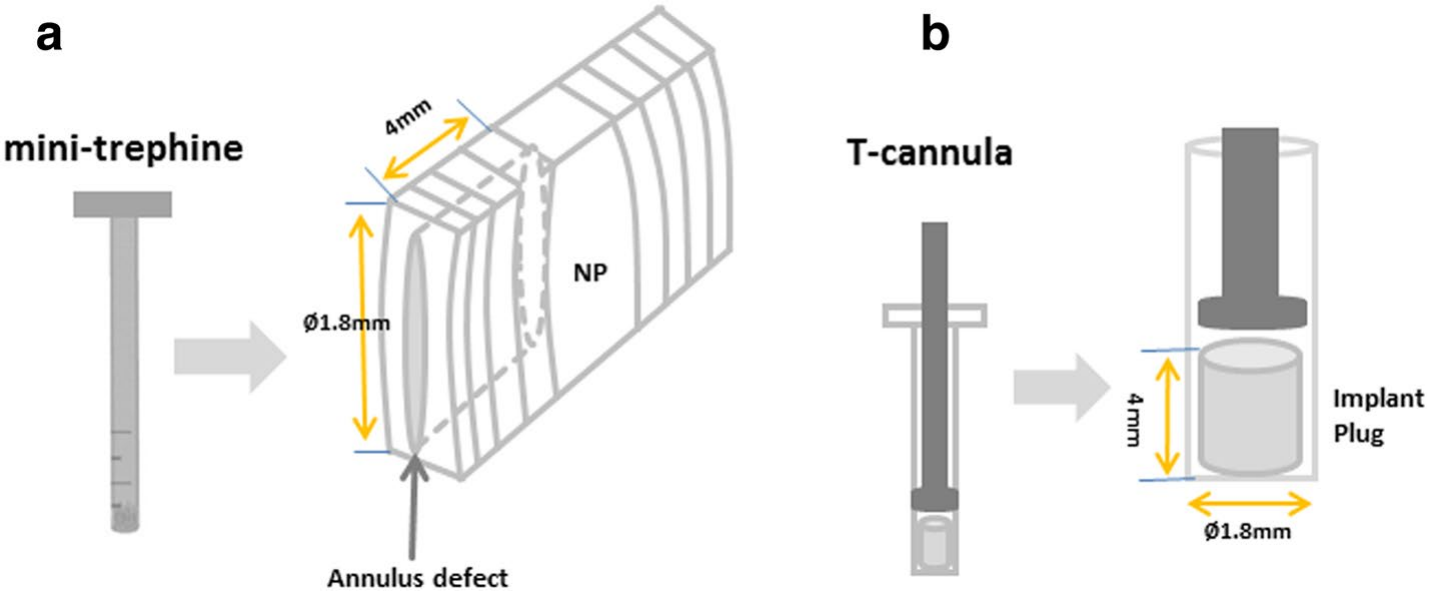
Schematic illustration of experimental instruments. The defect (Ø 1.8 mm; depth 4 mm) was created in the annulus wall using a mini-trephine. The depth of drilling was controlled by a *scale mark* (**a**); a delivery T-cannula was used for the implantation by press-fitting (**b**)

Two custom-made instruments were used, i.e., a mini-trephine with an external diameter of 1.8 mm and a delivery T-cannula, as illustrated in Fig. 1. To achieve a minimally invasive annulotomy, a defect (diameter 1.8 mm; depth 4 mm) was created in the annular wall using the mini-trephine. The depth of drilling was controlled by a scale mark (Fig. 1a). During the repair procedures, in order to achieve the initial stability of the implant placement and fill in the AF defect, a delivery T-cannula (inner diameter 1.8 mm) was used for the implantation by means of the press-fit technique. The implant was easily pushed out of the cannula into the annular defect (Fig. 1b). This technique guaranteed a tight fit between the implant plug and the defect.

### Implant preparation

The porous three-dimensional scaffold was fabricated as previously described [36]. Gelatin particles (280–450 μm) sieved from raw gelatin were added to a glass mould consisting of a cylindrical vial with a diameter of 22 mm. The vial was gently tapped and the surface of gelatin particles slightly pressed. The vial was then carefully moved into a vessel filled with saturated water vapour at 70 °C. After 1.5 h, the vial was removed, and the protuberant top surface caused by the swelling of the gelatin particles was immediately pressed flat. After cooling, 0.8 mL of PLGA/1, 4-dioxane solution (0.12 g/mL) was added drop by drop onto the gelatin-particle assembly. The mould was then maintained under a low pressure of 0.07–0.08 MPa to release air bubbles. Subsequently, the pressure was released to allow the polymer solution to fill the cavities among the gelatin particles. The mixture was frozen at −20 °C for 3 h, then freeze-dried to remove the 1, 4-dioxane. The porous PLGA scaffolds were obtained by leaching the gelatin assembly in 100 mL of deionized water at 70 °C for 10 h. The scaffolds had a micropore structure (ranging from 280 to 450 μm) and the pores were interconnected. The PLGA sponges were made into plugs of 1.8 mm diameter and 4 mm length. The scaffolds were sterilized by ethylene oxide.

### Surgical technique

**Fig. 2:**
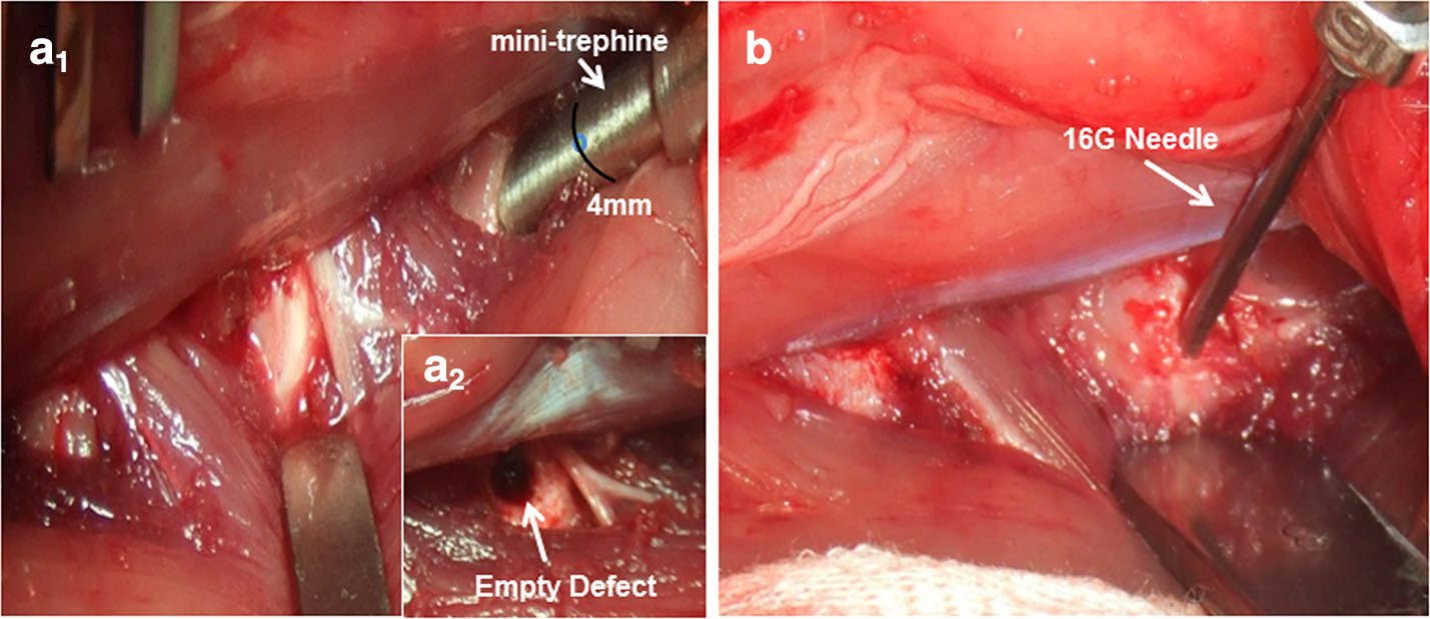
Surgical technique. Exposure of three consecutive intervertebral discs (L3/4, L4/5 and L5/6); creation of the defect using a mini-trephine (**a**_1_), empty defect (Ø 1.8 mm; depth 4 mm) in the anterolateral annulus (**a**_2_); puncture the annulus using a 16G needle to a depth of 5 mm (**b**). The *black ring* indicates a 4 mm depth

**Fig. 3:**
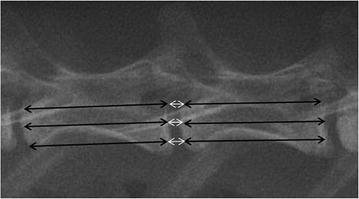
Disc height index (*DHI*) was calculated by average the IVD height (*white arrows*) and diving that by the average of adjacent vertebral body height (*black arrows*)

The rabbits were tranquillized by intramuscular injection of xylazine (3 mg/kg) and ketamine (40 mg/kg) and were then anaesthetized with sodium pentobarbital (30 mg/kg, Pharma Inc, Nanjing; PRC). All surgical procedures took place under aseptic conditions. The rabbits were placed on the operating table in a prone position. An anterolateral retroperitoneal approach was used to expose three consecutive levels of the rabbit IVD, comprising L3/4, L4/5 and L5/6. Experimental intervertebral discs were injured in the anterolateral AF. To exclude a spinal level bias, annular injuries were randomly allocated to four disc levels. The experimental discs were assigned to four groups: (1) Annulus trephination group: only creation of a defect in the annular wall. The mini-trephine (diameter 1.8 mm) was drilled into the AF and then rotated manually 360° within the disc. The final 4 mm depth of the drill channel was marked by a black ring (Fig. 2a_1_). The mini-trephine was then push backed to remove an annular cylinder from the drill site and a 4-mm-deep micro-hole (diameter 1.8 mm) was placed (Fig. 2a_2_); (2) Puncture group: annular puncture by 16G needle at a depth of 5 mm, as previously described [6, 7, 37] (Fig. 2b); (3) Implantation group: filling of the annular defect with a PLGA plug. The PLGA plug, prepared as described above, was carefully inserted through a delivery T-cannula into the empty defect and flushed with annular surface by the press-fit fashion; (4) Intact group: the L2/3 disc served as uninjured control. Finally, the wound was closed in layers. Following surgery, the rabbits were permitted free cage activity and food and water ad libitum. No surgery-related complications or neurological symptoms were observed in any of the rabbit.

### Magnetic resonance Imaging and radiographic analysis

Magnetic resonance images (MRI) and lateral plain radiographs were performed under general anaesthesia (sodium pentobarbital, 30 mg/kg) at 0.5, 1, 3 and 6 months after surgery (*n* = 6, per time point). MRI examinations were performed using a 1.5-T Imager with a quadrature extremity coil receiver. Midsagittal T2-weighted images were obtained in the following settings: fast spin echo sequence with time to repetition (TR) of 3500 ms, time to echo (TE) of 100 ms, 320(h) × 256(v) matrix; field of view 260; and number of excitations 4; and slice thickness 2 mm with a 0-mm gap. The MRI scans were evaluated by 2 blinded observers using the Pfirrmann’s classification scores [38] based on changes in the degree and area of signal intensity: 1 = normal, 2 = Inhomogeneous structure, high signal intensity, 3 = moderate decrease in signal intensity, but slightly narrowing the disc height, 4 = severe decrease in signal intensity, moderately narrowing the disc height. Lateral radiographs were obtained using a DR machine (General Electric Healthcare, Bucks, UK). The disc height was expressed as the disc height index (DHI), as previously described [6, 39]. The mean DHI was the ratio of the average measurements obtained from the anterior, middle and posterior portions of the IVD and the average of adjacent vertebral body heights (Fig. 3). Changes in the DHI were expressed as DHI % and normalized to the measured preoperative DHI (DHI % = postoperative DHI/preoperative DHI × 100). All measurements were done using the picture archiving and communication system (PACS) routinely used in the local hospital.

### Tissue harvesting

Six rabbits were euthanized by intravenous sodium pentobarbital overdose at 0.5, 1, 3 and 6 months after surgery. The experimental IVDs (L2/3, L3/4, L4/5 and L5/6), including approximately one-third of the adjacent vertebral bodies, were harvested from each lumbar spine under sterile conditions. The specimens were then dissected sagittally and divided into two symmetric parts. From one-half of each disc, the jelly-like nucleus pulposus (NP) was carefully separated from the AF and then snap-frozen in liquid nitrogen, with subsequent storage at −80 °C in preparation for PCR analysis. The other half was used for histology.

### qRT-PCR analysis

Total RNA was extracted from the pulverized NP tissues using TRIzol reagent (Invitrogen, Carlsbad, CA, USA) and purified using the RNeasy Mini Kit (Qiagen Inc). After extraction, RNA was quantified using a NanoDrop N-1000 spectrophotometer (Thermo Fisher Scientific, Wilmington, DE, USA). 1 μg of total RNA was reverse-transcribed into cDNA using the Superscript™ First Strand cDNA synthesis kit (Invitrogen, Carlsbad, CA, USA). The gene expression of aggrecan, type I collagen (Col1A1), type II collagen (Col2A1), MMP-3, glyceraldehyde-3-phosphate dehydrogenase (GAPDH) in the intervertebral discs were analysed by quantitative real-time PCR using Real-Time Detection System (Bio-Rad, hercules, CA, USA). All primer sequences are listed in Table 1. A positive standard curve for each primer was obtained using serially diluted cDNA sample mixture. Quantifications of gene expression for aggrecan, Col1A1, Col2A1 and MMP-3 were calculated using standard curves and normalized to GAPDH in each sample, and then the expression of treated discs was normalized to control discs.

**Table 1:** Oligonucleotide primers for PCR amplification

Gene	Primer sequence (5′–3′)	Annealing temperature (°C)
GAPDH	Forward: ACTCTGGCAAAGTGGATG Reverse: TCCTGGAAGATGGTGATG	60
Aggrecan	Forward: GAGGTCGTGGTGAAAGGTGT Reverse: GTGTGGATGGGGTACCTGAC	62
COL1A1	Forward: AGGGCCAAGACGAAGACATC Reverse: AGATCACGTCATCGCACAACA	60
COL2A1	Forward: GGATAGACCCCAACCAAGGC Reverse: GCTGCTCCACCAGTTCTTCT	62
MMP-3	Forward: GCCAAGAGATGCTGTTGATG Reverse: AGGTCTGTGAAGGCGTTGTA	65

### Gross morphological observation and histological evaluation

The excised IVDs were cut into sagittal halves for observation of gross morphology at 6 months after surgery. After macroscopic evaluation, the specimens were fixed in 10 % formalin, decalcified in ethylenediamine tetraacetic acid (EDTA), and processed for paraffin sectioning. Blocks of tissue were embedded in paraffin and sliced into 5-μm sections using a microtome. The sections were stained with hematoxylin and eosin (H & E) to observe changes in the AF and adjacent tissue, or Safranin-O staining for the assessment of the proteoglycan content. All stained sections were analysed under an optical microscope (Leica Microscope, Wetzlar, Germany) at magnifications ranging from 40 to 400×.

### Statistical analysis

Data were expressed as mean ± standard error of the mean. Statistical analysis was performed using SPSS 18.0 software (SPSS Inc., Chicago, IL, USA). Significant differences in the radiograph measurements were analysed by repeated-measurement analysis of variance (ANOVA) and Fisher’s least significant difference (LSD) test. The effect of time after surgery was analysed with the Kruskal–Wallis test. Mann–Whitney *U* tests were used to compare degeneration for the MRI and gene expression data. A value of *P* < 0.05 was considered statistically significant.

## Results

### Gross morphology

**Fig. 4:**
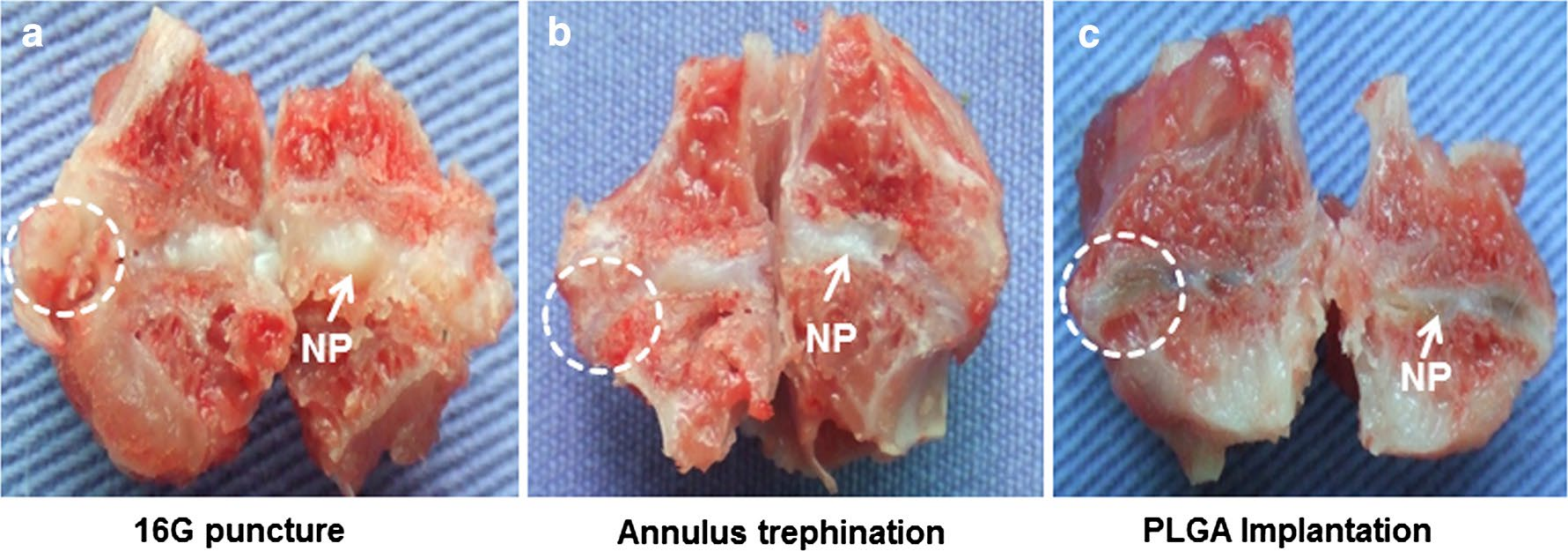
Gross morphology of the injured discs from the sagittal plane view at 6 months post-surgery. The 16G puncture group (**a**); the annulus trephination group (**b**); the PLGA implantation group (**c**). The *white circle* with a *dashed line* indicates the injury site

The gross appearance of the injured discs was observed by visual examination at 6 months post-surgery. In the 16G puncture group, a dense scar tissue appeared in the puncture site of the AF. The NP showed a loss of its shiny appearance and became less translucent (Fig. 4a). In the annulus trephination group, discernible disc narrowing and vertebral endplate deformity were clearly visible in the drilled annulus defect, with osteophyte formation around the edges of the vertebral body. The NP was less hydrated, more fibrotic-like changes (Fig. 4b). In contrast, in the PLGA implantation group, brown repair tissue filled in the defect and was well integrated into the surrounding annulus, while no obvious scar tissue was visible in the sagittal plane. The NP demonstrated a dull and less gel-like morphology (Fig. 4c).

### MRI and radiographic assessment after damage to the IVD

**Fig. 5:**
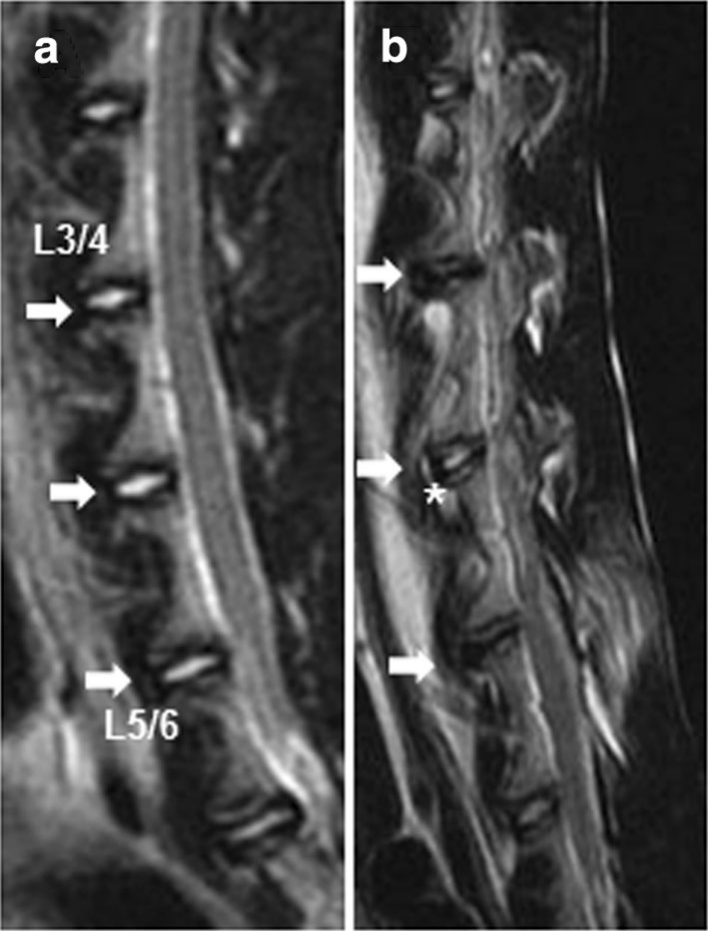
Representative magnetic resonance image (*MRI*) of the discs before surgery (**a**) and after surgery (**b**). Significant low T2-weighted signal intensity was clearly visible at the three experimental levels after surgery. The *white asterisk* indicates the partial loss of T2 signal intensity. The *arrows* indicate the operated disc

**Fig. 6:**
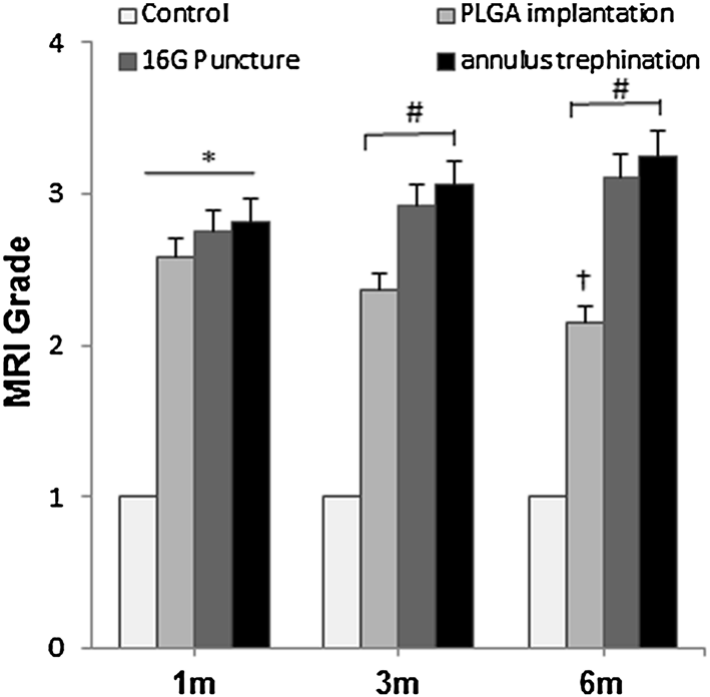
Changes in Magnetic resonance imaging (*MRI*) grade after surgery. The MRI grades of the three experimental groups after surgery were significantly higher than those of the control group. In addition, the MRI grade gradually increased in both the 16G puncture and annulus trephination group over the follow-up period (*P < 0.01, vs. control group). The MRI grade of the PLGA implantation group significantly decreased at 6 months post-surgery (^†^ *P* < 0.05, vs. 1 and 3 months). The MRI grade of the PLGA implantation group was lower than that of the 16G puncture group or annulus trephination (^#^ *P* < 0.05, vs. 16G puncture group or annulus trephination group)

Before surgery, the T2-weighted MRI appearance of the intact discs showed a relatively homogeneous, high signal intensity. 6 months after surgery, the NP of all operated discs showed a progressive decrease of signal intensity. As a matter of fact, the operated discs that received the PLGA implants showed a less pronounced loss of T2 signal intensity compared to the signals observed after 16G puncture or annulus trephination alone (Fig. 5a, b). In terms of semi-quantitative MRI scores, induction of IVD degeneration by all types of injury was associated with significantly higher MRI scores compared to the control group. In addition, the MRI scores increased gradually in both the 16G puncture and annulus trephination group over the follow-up period (*P* < 0.01). The MRI scores in the annulus trephination group was higher than in the 16G puncture group; however, this difference did not attain statistical significance (*P* > 0.05). At 1 month after surgery, the MRI scores of the PLGA implantation group did not significantly differ from those of the 16G puncture group or annulus trephination group; however, significant differences became evident at 3 and 6 months (*P* < 0.05), i.e., the PLGA implantation group showed a significantly lower MRI score compared with either the 16G puncture group or the annulus trephination group (*P* < 0.05) (Fig. 6).

**Fig. 7:**
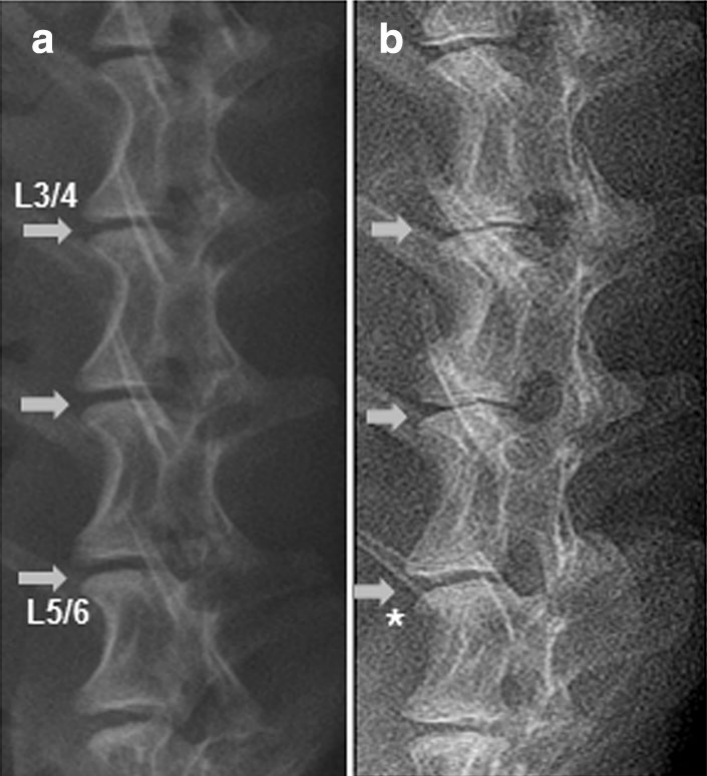
Representative lateral radiographs before surgery (**a**) and 6 months after surgery (**b**). Three experimental levels showed obvious and various degrees of disc space narrowing after surgery. Osteophyte formation (*white asterisk*) was seen at the margin of the vertebral bodies. The *arrows* indicate the operated disc

**Fig. 8:**
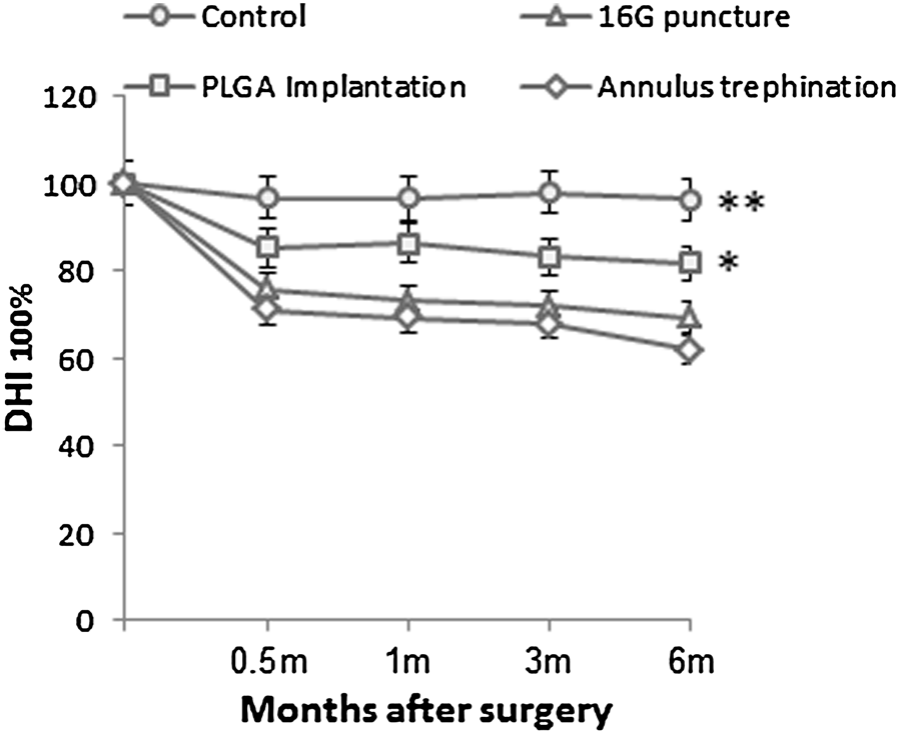
Changes in disc height index (*DHI*) after surgery. There was a slow, progressive decrease in the disc height of operated discs over time. The DHI in both the 16G puncture and annulus trephination groups were significantly lower than the DHI of the control group after surgery (**P < 0.01, vs. control group). The DHI of the PLGA implantation group was comparable to that of the control group and significantly higher than those in the 16G puncture group and annulus trephination group over the 6 months (*P < 0.05, vs. 16G puncture group or annulus trephination group)

Lateral radiographs of the spine before surgery showed a normal height of the disc space between each vertebra (Fig. 7a). In contrast, 6 months after surgery, there was a significant disc space narrowing of the operated discs, whereas osteophyte formation was visible at the margin of the vertebral bodies (white asterisk in Fig. 7b). In the control discs, no significant differences in disc height index (DHI) were observed at 6 months post-operation. In contrast, there was a slow, progressive decrease of disc height in the operated discs, and this was sustained for 6 months. The DHI in the 16G puncture and annulus trephination group decreased markedly at 0.5 month after surgery (and subsequently at a slower rate), and was significantly lower than that of the control group (*P* < 0.01). The degree of changes was similar for both groups (*P* > 0.05). In contrast, at 6 months post-surgery, the DHI of the PLGA implantation group was comparable to that of the control group. Notably, the DHI of the PLGA implantation group was significantly higher than those in the 16G puncture group and annulus trephination group at 1-month post-surgery and thereafter (*P* < 0.05) (Fig. 8).

### Real-time PCR

**Fig. 9:**
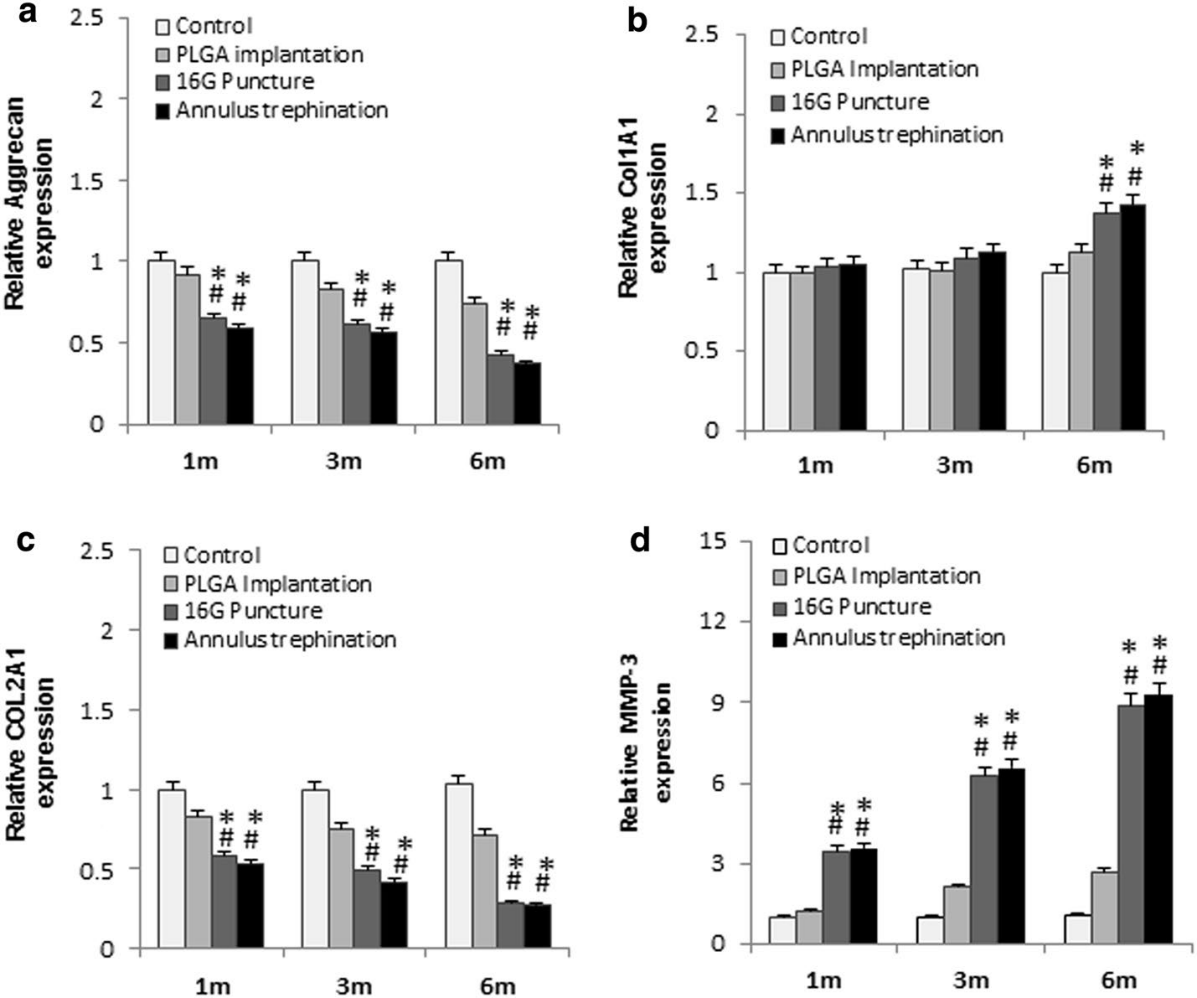
mRNA expression of aggrecan, Col1A1, Col2A1, MMP-3, at 1, 3, 6 months after surgery. The *graph* showed a marked reduction in aggrecan (**a**), Col2A1 (**c**), and significant increase in Col1A1 at 6 months post-surgery (**b**), and significant up-regulation of MMP-3 expression (**d**) in the injured discs, compared with the control group or the PLGA implantation group (*P < 0.01, vs. control group; # *P* < 0.01, vs. PLGA implantation group)

When compared with the control and PLGA implantation group, the expression of aggrecan, Col2A1 was significantly lower, and the expression of MMP-3 was markedly upregulated in both the 16G puncture group and annulus trephination group from 1 to 6 months after surgery (*P* < 0.01, Fig. 9a, c, d). The expression of Col1A1 increased significantly at 6 months post-surgery (*P* < 0.01, Fig. 9b). There were no significantly differences in the expression of aggrecan, Col1A1, Col2A1 and MMP-3 between two groups (*P* > 0.05) (Fig. 9a–d).

### Histological assessment

**Fig. 10:**
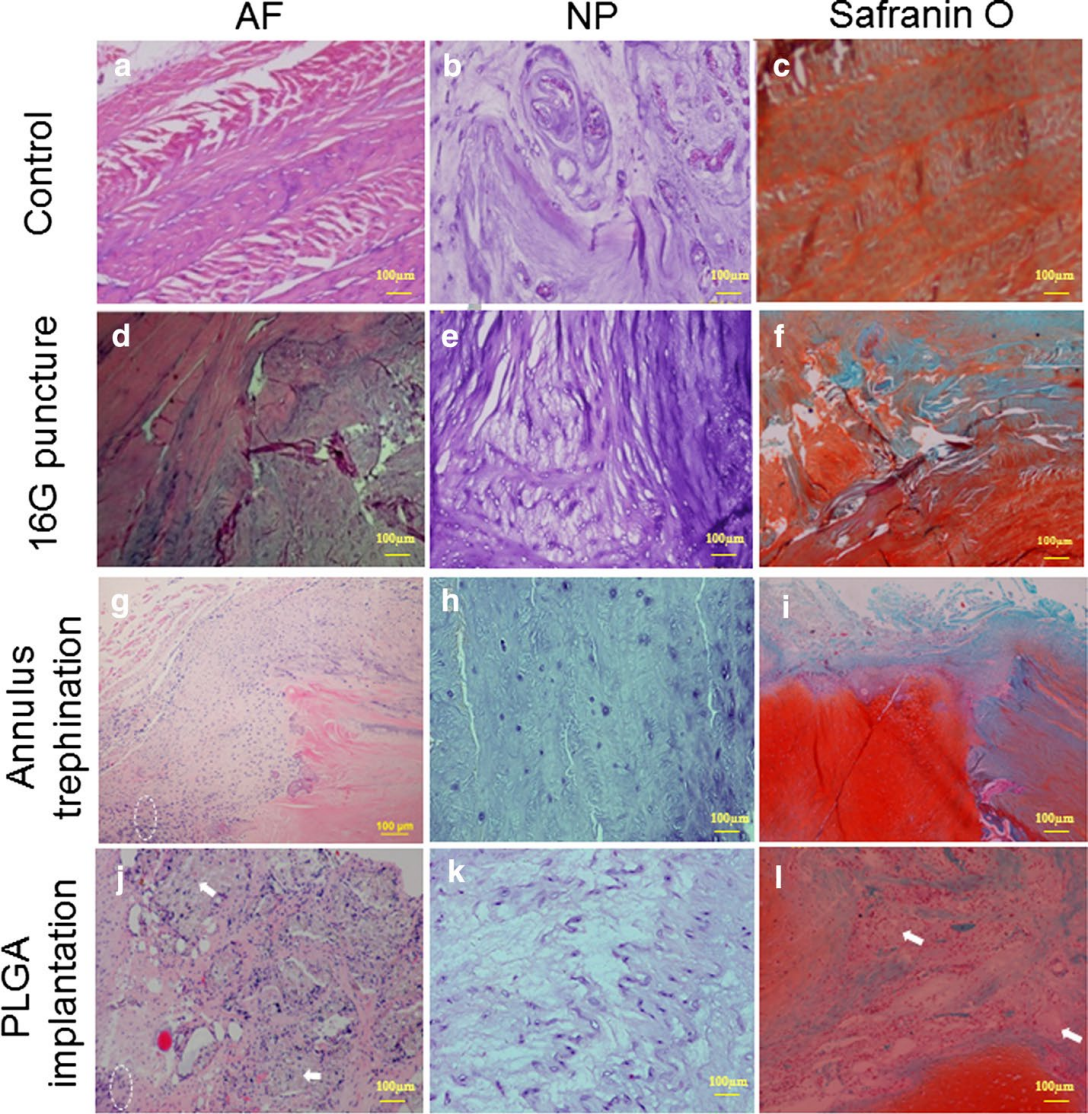
Hematoxylin/eosin (**a**, **b**, **d**, **e**, **g**, **h**, **j** and **k**) and Safranin O (**c**, **f**, **i** and **l**) staining of the annulus fibrosus (*AF*) and nucleus pulposus (*NP*) 6 months after surgery. Intact AF displayed a normal pattern of fibrocartilage lamellas with rich proteoglycans, as shown by strong Safranin O staining (**a**, **c**). The NP exhibited clusters of large, vacuolated cells (**b**). A collapsed, wavy appearance with less safranin O staining was evident at the puncture site (**d**, **f**). No vacuolated cells were detected in the NP, with increasingly chondrocyte-like cells (**e**). Annular defect was filled in extensive fibrocartilaginous tissue with rich proteoglycans; a distinct border was discernible between the filled tissue and the native annulus (**g**, **i**). The NP exhibited fewer chondrocyte-like cells (**h**). Clusters of new connective tissue were formed in original defects with small amounts of undegraded PLGA material. The proteoglycan content was markedly reduced in the repair tissue, except for a strongly safranin O stained region at the sites of cartilaginous tissue (**j**, **l**). The NP showed a slight proliferation of chondrocyte-like cells (**k**). *White arrows* indicate the undegraded PLGA; *white circle* with *dashed line* indicates the site of fibrocartilaginous tissue

At 6 months, H&E staining showed that the intact AF (control group) was characteristically well organized with its multilamellar structure. The safranin O staining indicated the presence of proteoglycan-rich matrix in the annulus. The NP exhibited clusters of large, vacuolated (notochordal) cells and smaller, chondrocyte-like cells (Fig. 10a–c). In the 16G puncture group, the annulus has a remarkably wavy, collapsed lamellar structure. Some regions of endochondral ossification and numerous small fissures were apparent at the annulus injury site. Loss of proteoglycan content with less pronounced safranin O staining was observed in the puncture track. No notochordal cells were detected in the NP, which was increasingly occupied by disorganized, chondrocyte-like cells (Fig. 10d–f). In the annulus trephination group, HE staining showed that the annular defect without lamellar structure was filled by extensive fibrocartilaginous-like tissue, indicating the signs of chondrogenesis. A well-defined border was discernible between the filled tissue and the adjacent native annulus. In addition, safranin O staining revealed a rich proteoglycan content in the fibrocartilaginous tissue. The NP exhibited further decrease in number of chondrocyte-like cells (Fig. 10g–l). In the PLGA implantation group, the progressive degradation of the original PLGA plug was simultaneous accompanied by new, immature repair tissue filling the defect. Small amounts of non-degraded PLGA material were surrounded by clusters of regenerated connective tissue. Poor safranin O staining indicated that the proteoglycan content was markedly reduced in the regenerated tissue, except for dense proteoglycan (strong staining region) at the sites of cartilaginous tissue. The NP exhibited a further slight proliferation of chondrocyte-like cells (Fig. 10j–l).

## Discussion

To the best of our knowledge, there is limited published information on minimally invasive annulotomy for models of IVD degeneration in the rabbit. In the present study, an animal model for the test and/or treatment of disc degeneration was successfully established in the rabbit. Partial removal of the AF with a 4 mm depth using a trephination technique is a relatively minor intervention to safely perform a less invasive ventral annulotomy. Similarly to previous reports [6–8, 15], our results showed that the disc injured by minimal trephination had significant decreases of DHI on lateral radiograph and high degeneration scores at MRI scans over the course of post-surgery observation, as confirmed by gene expression analysis, gross morphological and histological evaluation. In contrast, implantation of a PLGA plug reduced the negative impact of the experimental discs by reducing the loss of signal intensity at MRI and by significantly counteracting the progressive narrowing of the disc space.

So far, annular injury is one of the most common surgical modalities in various animal models of disc degeneration [4]. Previous studies have used several diffuse annular incisions, including a cruciate, rectangular excision, leading to extensive disruption of disc integrity [19, 20]. Further, the artificial setup of annular injury models can induce an acute NP herniation and is associated with a relatively rapid disc degeneration [40]. In particular, inappropriately directed needles or treatment can result in surgery-related complications, such as significant bleeding, nerve root injury, self-mutilation of a dysesthetic area or undesirable osteophyte [10, 41, 42]. Annular disruption may also lead to post-surgery leakage of intradiscal treatments or delivering materials through the annular fissure [43]. In an attempt to minimize the damage to the annular integrity and to easily accomplish a minimally invasive annulotomy, we specifically and successfully designed a mini-trephine that created a defect by simply removing the AF in the annular injury model. Notably, the annulotomy-induced rabbit model proved successful also in mimicking a slow progression of the degenerative process of the IVD. The present approach proved also quite safe, as no surgery-related complications were observed in any of the rabbits. Compared to the classical puncture approach, the results show that a minimal trephination may provide a reliable method of annular injury with limited surgical trauma to the annulus, well-suited for experimental studies on disc degeneration.

A concern arises that a critical size (diameter 1.8 mm; depth 4 mm) was applicable to create the defect in the annular wall in the present study. Since quantitative measurements of the rabbit disc have been performed on disc height (1.42 ± 0.39 mm) and disc height distribution (DHD, ranging from 1.87 to 2.01 mm) in thicker peripheral region than the centre. In addition, the measurement of anterior annulus thickness was 2.8 ± 0.30 mm [44, 45]. Several authors [46–48] reported that the size of a needle puncture in the annulus was in proportion with a disc’s mechanical property changes and closely related to disc degeneration. Based on the above-mentioned results, we think that the least disruptive annulotomy using an appropriately sized mini-trephine (diameter 1.8 mm) may preferably generate the anterior annular micro-defect, and meanwhile avoid the damage to adjacent structures leading to a relatively rapid disc degeneration. Accordingly, a 4 mm depth was served for surgical annulus trephination in the present study. Traditionally, it is considered a 5 mm depth of annular injury as standard approach for animal experiments in the smaller IVDs [49]. But a 5 mm depth could be too deep, causing a through-and-through lesion. Our results showed that significant disc degenerative changes occurred at each post-operative time point in the annular defect (diameter 1.8 mm, 4 mm depth) group, especially when compared between the 16G puncture and control group. Finally, the results support the hypothesis that minimally invasive annulotomy utilizing the appropriately sized mini-trephine may induce continuously progressive degenerative changes in the rabbit model. Importantly, it may also be an attractive alternative for studying the AF regrowth therapies, such as annular reconstruction in a small disc degeneration model.

Recent several studies [19, 50–54] have been introduced to repair annular defect by biological therapeutic approach and maintain the disc integrity. PLGA constructs with exhibiting a promising result of implantation into the degenerated disc to retard progressive degeneration in previous studies [26, 28, 31]. However, one potential drawback is that different implanted materials may be unsatisfactorily anchored in the annular defects. Furthermore, some animal experiments showed a high extrusion rate ranging from 20 to 33 % with a tissue engineered implantation [34, 43, 55, 56]. To retain the biomaterials in the repair region and support the regenerated tissue, it is critical that implanted material may be able to strongly adhere and be fully integrated with the native annulus tissue. In the current study, a custom-made T-cannula (diameter 1.8 mm) was deployed for pushing out the PLGA plug and subsequently closed the defect by press-fitting. As a result, initial stability of the PLGA implant is preferable to maintain by a tight fit between the PLGA plug and the defect. Compared with two untreated groups, the results of MRI and radiographic examination in the repair discs showed that PLGA plugs provided the mechanical stability and reduced the disc space narrowing. No early-term extrusions were observed in any of cases after post-operative intradiscal load. Lastly, this approach reduced the risk of implant extrusion in the present study. At 6 months post-implantation, histology further showed that immature regenerated tissue was formed in the original defect and PLGA material was already well integrated into the surrounding annulus without discernible borders. In contrast, the empty defect was filled in the fibrocartilage-like tissue, indicating a sign of chondrogenesis; this is in good agreement with previous studies [17, 25, 51, 57]. The results support our hypotheses that therapeutic PLGA plugs may improve the reparative capacity of AF within the degenerated IVDs and slow down disc degenerative process in the rabbit spine model.

However, this study has several limitations. Firstly, dealing with annular treatment in which generation and reconstruction of the defect were conducted at the same time. Annular repair was performed in an acute setting, which does not represent the chronic disc degeneration. Secondly, due to individual’s disc variations, imprecise trephination is a feasible way to increase the risk of impairing the endplate and reduce nutrient supply to disc. Thirdly, being a significant anatomic and biomechanical difference comparable with human spine, rabbits differed the properties of AF and degenerative situation. Thus, a larger animal model (i.e. porcine spine model) may represent more closely to the human situation, and further evaluation of the PLGA construct on annular repair would be beneficial. In addition, a relatively short observation period after surgery may obtain a less histological change of regenerated AF. In further studies, more efforts should be taken to heal the annular defect using the optimal therapeutic agents combined with multiphasic polymeric constructs in the chronic disc degeneration models.

## Conclusions

In summary, the minimally invasive approach for the creation of annular defects by an appropriately sized mini-Trephine is a suitable option for studying disc degeneration in a rabbit model. Implantation of a suitable PLGA plug induced a successful repair of the annulus fibrosus within the degenerated disc, and retarded the degenerative process in the annular injury model.
